# Role of Ultrasound in the Diagnosis of Chronic Kidney Disease and its Correlation with Serum Creatinine Level

**DOI:** 10.7759/cureus.4241

**Published:** 2019-03-12

**Authors:** Shakeel Ahmed, Sanobar Bughio, Maria Hassan, Sajan Lal, Muhammad Ali

**Affiliations:** 1 Urology, Sindh Institute of Urology and Transplantation, Karachi, PAK; 2 Radiology, Dr. Ziauddin Hospital, Karachi, PAK

**Keywords:** chronic kidney disease, renal echogenicity, serum creatinine

## Abstract

Objective: We aimed to study the role of ultrasound in the diagnosis of chronic kidney disease (CKD) and its correlation with serum creatinine level.

Materials and methods: This was a retrospective cross sectional study conducted in the ultrasound department of Dr. Ziauddin Hospital Clifton campus, Karachi from April 6, 2017 to October 6, 2017 for a period of six months. A total of 200 patients with CKD and glomerular filtration rate (GFR) determined to be < 60 ml/min were included in this study. Blood tests were gathered from the chosen patients, serum creatinine estimation was done for those patients and they underwent ultrasonography on the same day to assess echogenicity, parenchymal thickness, cortical thickness, and longitudinal length. This information was noted in the pro forma.

Results: The average age of the patients was 54.62±13.3 years. Mean serum creatinine was significant among echogenicity grades [p=0.0005]. Mean parenchymal thickness was also significant among echogenicity grades (p=0.0005). Mean longitudinal length was also significant among echogenicity grades (p=0.0005). Mean corticomedullary distinction was also significant among echogenicity grades (p=0.0005). A statistically significant highly positive correlation was observed between serum creatinine and cortical echogenicity grading (r=0.915 P = 0.0005).

Conclusion: The best sonographic parameter that correlates with serum creatinine is renal cortical echogenicity and its grading in comparison to longitudinal length, parenchymal thickness, and cortical thickness in patients of CKD. Since renal cortical echogenicity has the advantage of being irreversible in comparison to serum creatinine levels, it can be used as a parameter of renal function.

## Introduction

A deranged creatinine level over a period of few months to years is termed chronic kidney disease (CKD). CKD is based on the extent of kidney damage, calculated through decreased glomerular filtration rate (GFR) (i.e. < 60 ml/min per 1.7 m^2)^ for more than three months [1, 2].

Ultrasonography is a noninvasive and inexpensive investigation modality with sufficient anatomical details necessary to diagnose renal diseases without exposing the patient to radiation or contrast and hence has replaced standard radiography in our country and abroad [3-5]. All these factors promote early detection and prediction of deranged renal function tests necessary for making a therapeutic decision.

Sonography identifies renal length, thickness, and echogenicity of renal parenchyma apart from its importance in detailing a dilated collecting system [6]. These details assist in identifying the extent of renal parenchymal damage and the possibility of its reversibility [7, 8], and the decision to perform a renal biopsy [9]. According to a study, abnormal sonographic findings were seen in 67% of cases of CKD [10].

Due to the presence of collagen, echogenicity is increased in interstitial fibrosis and glomerulosclerosis [11], but this has never been recognized. Increase in echogenicity may also increase interstitial inflammation. The human eye can also assess echogenicity but it is unreliable [12, 13]. In a small group of adults, renal parenchymal echogenicity can be reliably quantitated and established within a normal range [14]. It was found that there is significant correlation between renal length or cortical echogenicity with glomerular sclerosis or tubular atrophy [15].

Renal morphology can be determined by a number of means that include measuring renal length and volume and renal cortical thickness. Renal function can also be evaluated through renal length and cortical thickness, and important clinical decisions can be made on its basis. Therefore serial sonographic evaluations are done to find out the progression of renal disease or its normality [16]. Although renal parenchymal volume is quite an accurate measurement in patients with end stage renal disease, measurement of renal longitudinal length is sufficient in normal patients [17].

Hence, ultrasound is a good modality to ascertain renal insufficiency and progression of disease. The aim of our study was to correlate renal echogenicity with serum creatinine levels and to investigate the significance of renal echogenicity in identifying the progression of CKD as well as the use of sonographic imaging in the grading of CKD.

## Materials and methods

This was a retrospective cross-sectional study conducted in the ultrasound department of Dr. Ziauddin Hospital Clifton campus, Karachi from April 6, 2017 to October 6, 2017 for a period of six months. All patients referred for an ultrasound of the kidneys, whose creatinine was checked on the same day on which the ultrasound was performed, were considered. A total of 200 patients were included in this study.

New patients presenting for CKD workup, patients known to have CKD as per operational definition, CKD stages 3/4/5 and GFR determined to be < 60 ml/min as calculated by the Modification of Diet in Renal Disease (MDRD) equation, and patients of age above 30 years (male and female) were included in the study. Known patients of acute kidney injury, kidney transplant patients, patients on hemodialysis, patients on peritoneal dialysis, patients with fatty liver, chronic liver disease and solitary kidney were excluded from the study.

Ultrasound of the kidneys and liver was performed using the standard B Mode grey scale ultrasound with sector curved array transducer of 3.5-5 MHz. The parenchymal echogenicity of both the liver and kidney was assessed by applying low tissue harmonic and speckle reduction imaging to reduce the interobserver bias. The gain and time gain compensation were adjusted manually. The longitudinal length was measured in a section visually estimated to represent the largest longitudinal section. The width and thickness were measured in a section perpendicular to the longitudinal axis of the kidney as assessed from the longitudinal image. It is not necessary to keep the ultrasound probe perpendicular to the skin. However, the level of this transverse section was placed quite close to the hilum of the kidney but at the same time free of the pelvis.

Statistical data analysis was done using the Statistical Package for the Social Sciences (SPSS version 20) (IBM Corp., Armonk, NY). Mean and SD were calculated for age; mean parenchymal thickness, mean longitudinal size, frequency and percentages were calculated for gender and grade of echogenicity. Statistical analysis was calculated using one way analysis of variance (ANOVA). The relationship between serum creatinine and sonographic parameters was assessed by correlation coefficient analysis. P values less than 0.05 were considered statistically significant.

## Results

A total of 200 patients with CKD and GFR determined to be < 60 ml/min as calculated by the MDRD equation were included in this study. Twenty percent of the patients were below and equal to 40 years of age, 42.5% were between 41 and 60 years, and 37.5% were above 60 years of age. The average age of the patients was 54.62 ± 13.3 years; the average serum creatinine, parenchymal thickness, longitudinal length, and cortical thickness are shown in Table 1.

**Table 1:** Descriptive Statistics of the Characteristics of the Patients [n=200]

Variables	Mean ± SD	95% Confidence Interval for Mean		Median (IQR)
		Lower Bound	Upper Bound	
Age (years)	54.62±13.30	52.77	56.47	56 (21)
Serum creatinine (mg/dl)	2.19±1.08	2.046	2.337	1.9 (1.3)
Parenchymal thickness	4.69±0.82	4.578	4.806	4.5 (0.9)
Longitudinal length	9.87±0.94	9.745	10.008	10 (0.8)
Cortical thickness	0.94±0.253	0.902	0.973	1.1 (0.3)

**Figure 1:**
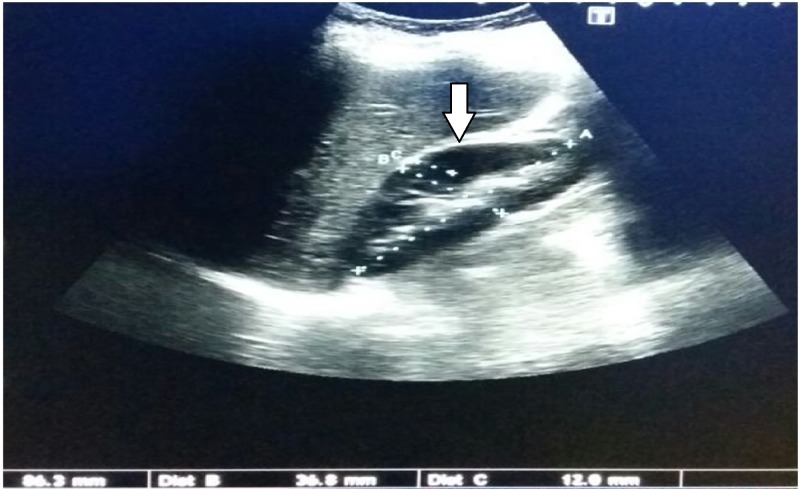
Grade-0 Echogenicity of Renal Parenchyma Less Than That of the Liver with Maintained Corticomedullary Distinction

Out of the 200 patients, 60% were male and 40% were female. The ultrasound findings of the renal parenchymal changes and its grading, Grade 0 is shown in Figure 1.

Sixty (30%) patients had Grade 0 parenchymal changes, 60 (30%) patients had Grade 1 parenchymal changes, 44 (22%) patients had Grade 2 parenchymal changes, 24 (12%) patients had Grade 3 parenchymal changes, and 12 (6%) patients had Grade 4 parenchymal changes.

The mean serum creatinine was 1.252±0.050 mg/dl (95%CI: 1.239 to 1.265) for Grade 0, 1.853±0.129 mg/dl (95%CI: 1.82 to 1.887) for Grade 1, 2.568±0.651 mg/dl (95%CI: 2.370 to 2.766) for Grade 2, 3.275±0.352 mg/dl (95%CI: 3.126 to 3.424) for Grade 3, and 5.033±0.528 mg/dl (95%CI: 4.698 to 5.369) for Grade 4.

Mean serum creatinine was significant among echogenicity grades [ANOVA F-Value= 367.726; p=0.0005] (Table 2).

**Table 2:** Comparison of Serum Creatinine with Renal Cortical Echogenicity

Echogenicity Grades [Grading based on ultrasound features]	Serum Creatinine (mg/dl)						
	No. of Patients	Mean	SD	95% Confidence Interval for Mean		F Value	P-Value
				Lower Bound	Upper Bound		
Grade 0	60	1.252	0.050	1.239	1.265	367.726	0.0005
Grade 1	60	1.853	0.129	1.820	1.887		
Grade 2	44	2.568	0.651	2.370	2.766		
Grade 3	24	3.275	0.352	3.126	3.424		
Grade 4	12	5.033	0.528	4.698	5.369		
Total	200	2.191	1.0431	2.046	2.337

Mean parenchymal thickness was also significant among echogenicity grades (ANOVA F-value= 31.628; p=0.0005) (Table 3).

**Table 3:** Comparison of Parenchymal Thickness with Renal Cortical Echogenicity

Echogenicity Grades [Grading based on ultrasound features]	Parenchymal Thickness (cm)						
	No. of Patients	Mean	SD	95% Confidence Interval for Mean		F Value	P-Value
				Lower Bound	Upper Bound		
Grade 0	60	4.397	0.101	4.371	4.423	31.628	0.0005
Grade 1	60	4.582	0.905	4.348	4.816		
Grade 2	44	5.048	0.920	4.768	5.328		
Grade 3	24	4.433	0.481	4.230	4.637		
Grade 4	12	5.933	1.029	5.280	6.587		
Total	200	4.692	0.820	4.578	4.806

Mean longitudinal length was also significant among echogenicity grades (ANOVA F-value= 66.004; p=0.0005) (Table 4).

**Table 4:** Comparison of Longitudinal Length with Renal Cortical Echogenicity

Echogenicity Grades [Grading based on ultrasound features]	Longitudinal Length (cm)						
	No. of Patients	Mean	SD	95% Confidence Interval for Mean		F Value	P-Value
				Lower Bound	Upper Bound		
Grade 0	60	10.000	0.0000	10.000	10.000	66.004	0.0005
Grade 1	60	10.272	0.8114	10.062	10.481		
Grade 2	44	10.348	0.7457	10.121	10.574		
Grade 3	24	8.758	0.4898	8.551	8.965		
Grade 4	12	7.792	0.8107	7.277	8.307		
Total	200	9.876	0.9450	9.745	10.008

Mean cortical thickness was also significant among echogenicity grades (ANOVA F-value= 477.83; p=0.0005) (Table 5).

**Table 5:** Comparison of Cortical Thickness with Renal Cortical Echogenicity

Echogenicity Grades [Grading based on ultrasound features]	Cortical Thickness (cm)						
	No. of Patients	Mean	SD	95% Confidence Interval for Mean		F Value	P-Value
				Lower Bound	Upper Bound		
Grade 0	60	1.100	0.0000	1.100	1.100	477.83	0.0005
Grade 1	60	1.093	0.0756	1.074	1.113		
Grade 2	44	0.900	0.1034	0.869	0.931		
Grade 3	24	0.462	0.1135	0.415	0.510		
Grade 4	12	0.433	0.0888	0.377	0.490		
Total	200	0.938	0.2531	0.902	0.973

A statistically significant highly positive correlation was observed between serum creatinine and cortical echogenicity grading (r=0.915 P = 0.0005) as shown in Table 6. There was also a statistically significant positive correlation between mean parenchymal thickness and renal echogenicity (r=0.336; P 0.005). There was also a statistically significant negative correlation between longitudinal length, cortical thickness with renal echogenicity (r= -0.513; P= 0.005) and (r= - 0.869; P= 0.0005), respectively (Table 6).

A statistically significant negative correlation was also observed between longitudinal size and serum creatinine (r= -0.505; P = 0.0005); a statistically significant negative correlation was observed between cortical thickness and serum creatinine (r= - 0.845; P = 0.0005) and a statistically significant positive correlation was observed between parenchymal thickness and serum creatinine (r=0.413; P = 0.0005) (Table 6).

**Table 6:** Statistical Correlation between Serum Creatinine and Mean Parenchymal Thickness, Mean Longitudinal Size, Mean Cortical Thickness and Echogenicity grade ** Correlation is significant at the 0.01 level (2-tailed).

		Parenchymal Thickness	Longitudinal Length	Cortical Thickness	Echogenicity Grade
Serum creatinine (mg/dl)	Pearson correlation	0.413**	-0.505**	-0.845**	0.915**
	P-Value	0.0005	0.0005	0.0005	0.0005
Parenchymal thickness	Pearson correlation		0.092	-0.240**	0.336**
	P-Value		0.194	0.001	0.005
Longitudinal length	Pearson correlation			0.634**	-0.513**
	P-Value			0.0005	0.0005
Cortical thickness	Pearson correlation				-0.869**
	P-Value				0.0005

## Discussion

The term chronic kidney disease means progressive damage to kidneys caused by structural or functional abnormalities of the kidney that can get worse over time. When the damage gets worse, the kidneys stop working, with or without decreasing GFR, and it is manifested by either pathological abnormalities or changes in markers of kidney damage or abnormalities in the imaging tests [18].

This study determines the functional capacity of the kidneys in CKD and determination of GFR using serum creatinine. Sonography is the ideal imaging modality as it is easily available and affordable to provide real-time information on the renal measurements and echogenicity.

Ultrasonographic findings such as longitudinal length, echogenicity, parenchymal and cortical thickness can be affected by chronic kidney disease [19, 20]. The GFR and stage of the disease can be ascertained by the endogenous serum creatinine level [21].

According to O’Neill, the useful upper limit of the normal range for kidney length is said to be 12 cm [20], whereas in our study the mean longitudinal length was 9.7 cm. According to Fiorini and Barozzi, renal length under 8 cm is definitely reduced and should be attributed to chronic renal failure [22]. As renal length decreases with decreasing renal function, renal length has traditionally been considered a surrogate marker of renal function. Hence for progression of disease process, estimation of renal length should be preferred to renal volume.

The mean serum creatinine in our study was 1.25 mg/dl for Grade 0, 1.85 mg/dl for Grade 1, 2.5 mg/dl for Grade 2, 3.27 mg/dl for Grade 3, and 5.03 mg/dl for Grade 4. Mean serum creatinine was significant among echogenicity grades [ANOVA F-Value= 367.726; p=0.0005]. Our study showed a statistically significant correlation between serum creatinine and the grade of echogenicity (p=0.0005 ). This value was similarly seen in a study by Siddappa et al. who also noted a statistically significant correlation between these parameters (p=0.004) [23]. Studies by Ibinaiye et al. and Singh A et al. [24] showed similar values (p=<0.001).

This study showed that the mean parenchymal thickness was significant among echogenicity grades (ANOVA F-value= 31.628; p=0.0005). Similar findings was also obtained in the study by Siddappa et al. There was a statistically significant positive correlation observed between renal echogenicity grading and parenchymal thickness (p = 0.009) [25]. As the echogenicity increased, there was a decrease in the mean parenchymal thickness.

This study showed that the mean cortical thickness was significant among echogenicity grades (ANOVA F-value= 477.83; p=0.0005). In our study the mean cortical thickness was found to be 9.3 mm (p=0.0005). Similar results were shown in the study by Singh A et al., where mean cortical thickness was found to be 8.5 mm [24]. As the echogenicity increased, there was a decrease in mean cortical thickness.

The limitation of this study is that this study group included more patients with Grade 1 and Grade 2 in comparison to patients with Grade 3 and Grade 4 CKD. This most likely occurred as this study was conducted in a tertiary care hospital, where most cases were treated with renal replacement therapies like hemodialysis, peritoneal dialysis, and renal transplantation, and such patients were excluded from this study.

As serum creatinine increases, the renal cortical echogenicity is increased. Since changes in renal echogenicity are irreversible, a sonological grading of CKD can be carried out, allowing the severity of CKD to be assessed.

## Conclusions

The best sonographic parameter that correlates with serum creatinine is renal cortical echogenicity and its grading in comparison to longitudinal length, parenchymal thickness and cortical thickness in patients of CKD. Since renal cortical echogenicity has the advantage of being irreversible in comparison to serum creatinine levels, it can be used as a parameter of renal function.
